# Multi-omic profiling of simultaneous ductal carcinoma in situ and invasive breast cancer

**DOI:** 10.1007/s10549-024-07270-5

**Published:** 2024-03-24

**Authors:** Henry G. Kaplan, Alexa K. Dowdell, Anna B. Berry, Racheli Ben Shimol, Fred L. Robinson, Christopher A. Carney, Brian D. Piening

**Affiliations:** 1 Swedish Cancer Institute, 1221 Madison St., Suite 920, Seattle, WA 98104 USA; 2 Earle A. Chiles Research Institute, Providence Health, Portland, OR 97213 USA

**Keywords:** DCIS, Genomics, Breast cancer, RNA-seq

## Abstract

**Purpose:**

The progression of ductal carcinoma in situ (DCIS) to invasive breast carcinoma (IBC) in humans is highly variable. To better understand the relationship between them, we performed a multi-omic characterization of co-occurring DCIS and IBC lesions in a cohort of individuals.

**Methods:**

Formalin-fixed paraffin-embedded tissue samples from 50 patients with co-occurring DCIS and IBC lesions were subjected to DNA-seq and whole transcriptome RNA-seq. Paired DCIS and IBC multi-omics profiles were then interrogated for DNA mutations, gene expression profiles and pathway analysis.

**Results:**

Most small variants and copy number variations were shared between co-occurring DCIS and IBC lesions, with IBC exhibiting on average a higher degree of additional mutations. However, 36% of co-occurring lesions shared no common mutations and 49% shared no common copy number variations. The most frequent genomic variants in both DCIS and IBC were *PIK3CA, TP53, KMT2C, MAP3K1, GATA3* and *SF3B1*, with *KMT2C* being more frequent in DCIS and *TP53* and *MAP3K1* more frequent in IBC, though the numbers are too small for definitive conclusions. The most frequent copy number variations were seen in *MCL1, CKSB1* and *ERBB2. ERBB2* changes were not seen in IBC unless present in the corresponding DCIS. Transcriptional profiles were highly distinct between DCIS and IBC, with DCIS exhibiting upregulation of immune-related signatures, while IBC showed significant overexpression in genes and pathways associated with cell division and proliferation. Interestingly, DCIS and IBC exhibited significant differential expression of different components of extracellular matrix (ECM) formation and regulation, with DCIS showing overexpression of ECM-membrane interaction components while IBC showed upregulation of genes associated with fibronectin and invadopodia.

**Conclusion:**

While most co-occurring DCIS and IBC were mutationally similar and suggestive of a common clonal progenitor, transcriptionally the lesions are highly distinct, with IBC expressing key pathways that facilitate invasion and proliferation. These results are suggestive of additional levels of regulation, epigenetic or other, that facilitate the acquisition of invasive properties during tumor evolution.

**Supplementary Information:**

The online version contains supplementary material available at 10.1007/s10549-024-07270-5.

## Introduction

The diagnosis of ductal carcinoma in situ (DCIS) has increased significantly with the introduction of screening mammography and now represents 20% of newly diagnosed breast cancer in the United States. DCIS may be found either with or without simultaneous invasive disease. Invasive ductal cancer presents with synchronous DCIS approximately 60% of the time [1]. Additionally, invasive breast carcinoma (IBC) may develop either without preexisting DCIS or temporally distant from earlier pure DCIS. While isolated DCIS has an excellent prognosis, patients with DCIS have an increased risk of invasive disease and breast cancer death, particularly at younger ages [2–6]. Studies have suggested that DCIS can evolve into invasive disease but the frequency of this process and whether or not DCIS and invasive disease may represent independent processes in the same breast are unclear [7].

A variety of studies have shown that genomic copy number variations both genome-wide and at specific loci are a prognostic factor for progression and/or recurrence for DCIS [8–11]. Moreover, gene expression studies have shown significant heterogeneity in DCIS expression profiles, with hallmarks of the major gene expression subtypes for IBC often already evident in DCIS lesions [12–16]. There also appears to be a complex interplay between DCIS progression and the tumor immune microenvironment (TIME), with tumor infiltrating lymphocytes (TIL) largely associated with higher-grade DCIS lesions but largely transitioning to an immune-excluded environment in IBC [17–19]. A variety of studies have also examined the genomics and transcriptomics of DCIS and IBC in co-occurring and non-co-occurring lesions to implicate a variety of genes potentially associated with progression [1, 20–23]. However, there remain gaps in our understanding of the key molecular processes associated with DCIS transformation and invasion. As such, we performed a multi-omic investigation of genomic and transcriptomic similarities and differences in a cohort of patients who presented with simultaneous DCIS and invasive breast cancer in the same quadrant of the same breast.

## Methods

### Patient recruitment

Patients were identified from the Swedish Cancer Institute (SCI) Breast Cancer Database starting sequentially with 2018 backwards to identify candidates with simultaneous DCIS and invasive ductal cancer. Individual pathologic review was performed from the cohort of patients who had had modified radical mastectomy to select 50 cases for analysis of normal breast, DCIS, and invasive disease.

### Molecular analysis

Formalin-fixed paraffin-embedded (FFPE) tissue blocks were retrieved from pathology archives. Ten 5 µm sections were prepared on microscopy slides for next-generation sequencing (NGS) analysis. DNA and RNA extraction, library preparation and sequencing were performed by Tempus Labs, Inc. (Chicago, Ill) using the Tempus xT workflow (DNA-seq of 648 genes at 500 × coverage), as previously described [24, 25]. DNA-seq alignment, mapping and variant calling were performed using the Tempus xT informatics pipeline. Clinically significant mutations identified by this assay include germline and/or somatic single nucleotide variants, insertions/deletions, and copy number variations, as well as structural rearrangements in a subset of 22 genes. Additionally, whole transcriptome RNA-seq was performed using the KAPA RNA HyperPrep kit. Tempus uses total RNA into library prep and exome hybrid capture to select the library. While the kits used across the two sample batches were the same, there was a change in adapters used for the second batch of RNA-seq, resulting in a difference in library strandedness. Both DNA-seq and bulk RNA-seq were performed in two batches.

### Informatics

Raw reads were demultiplexed using bcl2fastq v2.20 into FASTQ format. FASTQ files were aligned to GRCh38 GENCODE Human release 39 using STAR v2.7-10a. STAR run parameters were adapted from the ENCODE RNA-Seq pipeline for gene count quantification [26]. Strandedness between batches of sequencing was identified using the checkstrandedness function from how_are_we_stranded_here to ensure the correct read counts column was pulled for downstream analyses [27]. The counts were preprocessed by removing genes with less than an average of 5 counts across all samples. Differential gene expression analysis was performed on pairwise samples for patient cases who had both a DCIS and IBC sample sequenced using DESeq2 [28] with visuals created with ComplexHeatmap and EnhancedVolcano [29–31].

Two methods of pathway enrichment analysis were utilized. First, a pre-ranked gene list was generated from sorting the DESeq2 contrast results by log fold change for Gene Set Enrichment Analysis (GSEA) against MSigDB collections C2, C3 transcription factor targets, and C5 biological processes [32]. Gene sets were visualized using the EnrichmentMap app in Cytoscape [33]. Significantly enriched pathways were assessed at an FDR corrected *p*-value ≤ 0.1 with node color indicating the GSEA assigned normalized enrichment score (NES). Secondly, the DAVID functional annotation tool was used on the lists of differentially expressed genes at *q*-value ≤ 0.001 focusing on GO biological processes, KEGG, and Reactome pathway databases [34, 35]. Reactome Pathway Browser was utilized by inputting the lists of differentially expressed genes enriched for each side of the contrast separately to visualize pathway specific gene expression [36].

Samples excluded from downstream RNA analyses were due to a patient only having a single sample, RNA quantity not sufficient, failed sequencing quality control metrics, or failed DNA-seq data which was the case for a single patient for DNA-seq and RNA-seq integrative analyses. Whole transcriptome similarity between samples was assessed by calculating Euclidean distances on the variance stabilizing transformed DESeq2 object and the heatmap of sample-to-sample distances was created via pheatmap. Statistical tests comparing the mean Euclidean distance for patient’s pairwise samples whose DCIS and IBC shared clinically significant variants versus those that did not was conducted via Student’s *T*-test.

## Results

### Cohort characteristics

**Fig. 1:**
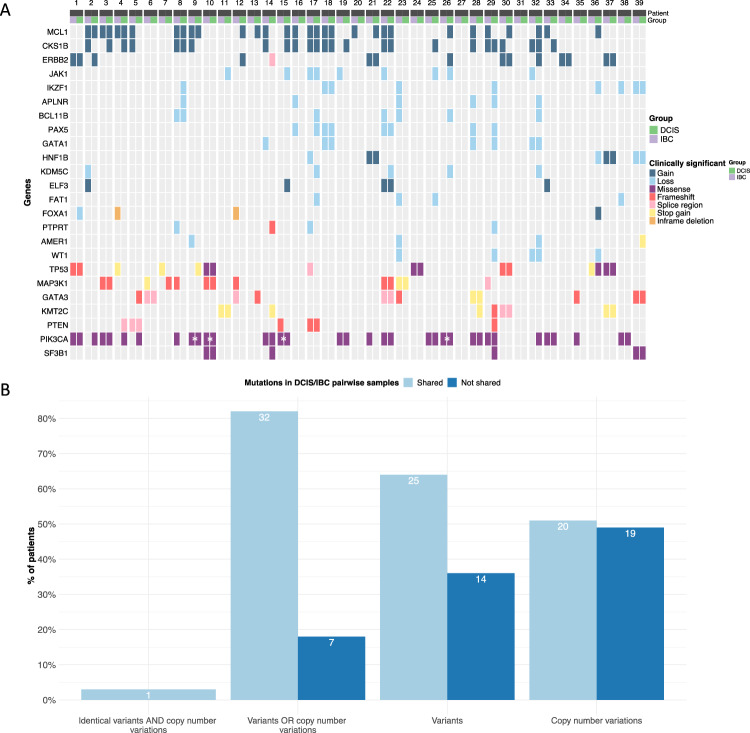
Global landscape of clinically significant variants and copy number variations across all 39 patients. **A** Discrete heatmap indicating presence or absence of variant and copy number variations (CNVs) for all 39 patients with paired DCIS and IBC DNA-seq results; see associated legend for the variant type color coding. White asterisks indicate the DCIS and IBC tumors for a given patient had mutations in the same gene but the mutation was different. **B** Barplot indicating the percentage of patients with shared genomic variants and copy number variations between DCIS and IBC tumors

We selected a series of thirty-nine cases of co-occurring DCIS and IBC in the same quadrant of the same breast with adequate tissue for both DCIS and IBC for DNA-seq. (Thirty-one of these cases also had adequate tissue RNA for RNA-seq.) 29/39 were estrogen receptor (ER)/progesterone receptor (PR) positive and *ERBB2* negative. 4/39 were ER + /PR-/*ERBB2* negative. 2/39 were triple positive, 3/39 were ER+ /PR-/*ERBB2* positive and 1/39 was ER/PR negative and *ERBB2* positive. 32/39 had Ki-67 ≤ 20% (IQR 14 (9, 19.5). 35/39 IBC were T1 or T2. Three were T3 and one T4. 21/39 IBC were N0, 4/39 N1_mic_, 9/39 N1a, 3/39 N2a, 2/39 N3a. Comprehensive genomic profiling using a 648-gene panel (Tempus xT) specimens and whole transcriptome RNA-seq were performed on all paired DCIS and IBC. The mutational profiles for the paired specimens across the most frequent alterations can be seen in Fig. 1A. There was a much higher degree of inter-patient variation than intra, with the majority of cases 25/39 (64%) sharing at least one reported small variant between DCIS and IBC (Fig. 1A, B). 14/39 (36%) had no shared variants (5 cases had no detected variants). 20/39 (51%) shared at least one copy number variation (CNV). 19/39 (49%) had no shared copy number variations (3 had no copy number variations). 32/39 (82%) shared a variant or copy number variation. 7/39 (18%) shared no variants or copy number variations while considering all patients who had at least one small variant or copy number variation. From the figure we can also see a high degree of variability across the cohort, with only 1/39 (3%) of patients exhibiting an identical mutational profile between DCIS and IBC.

Amongst the detected genomic variants, the most commonly mutated genes were quite similar between IBC and DCIS (Fig. 1A and Table 1). Despite this, no alterations were universal across the cohort, with even common driver alterations such as *PIK3CA* repeatedly exhibiting mutation in only one of a patient’s paired DCIS and IBC, perhaps suggestive of a non-clonal relationship between the lesions in these patients. For copy number variations (CNVs), we observed that the most frequently detected CNVs were in *CKS1B, MCL1* and *ERBB2* for both DCIS and IBC (Table 2). Moreover, CNVs were not always detected across both of a DCIS/IBC pair and *ERBB2* was not seen in IBC without its presence in the corresponding DCIS (Table 3). A total of 130 CNV occurrences were observed across 53 unique CNVs amongst all DCIS tumors (0–12 patient). Similarly, we observed 121 CNV occurrences across 51 unique CNVs within invasive tumors (0–9/patient). There were 43 instances where the same CNV was reported in both the DCIS and paired IBC tumors (0–5/patient). Additionally, 87 instances of CNVs were detected in DCIS but not paired IBC (0–11/patient) and 75 CNVs  were detected in IBC but not paired DCIS (0–9/patient). Furthermore, 73 unique small variants were observed across 94 variant occurrences within DCIS tumors (0–8/patient), while 76 unique variants were reported across a total of 87 variant occurrences within IBC tumors (0–6/patient). A total of 45 instances were observed where the same variant was detected in both the DCIS and paired IBC (0–4/patient) with an additional 44 instances of variants observed in DCIS but not paired IBC (0–5/patient), and 42 variants observed in IBC but not paired DCIS (0–5/patient). Tumor mutation burden was universally low across DCIS and IBC in this cohort with a median TMB of 2.1 (1.1, 3.2) for DCIS and 1.6 (1.1, 2.6) for IBC.

**Table 1:** Most frequent clinically significant variants in DCIS and IBC

Top variants	DCIS frequency (N)	IBC Frequency (N)
PIK3CA	20	18
TP53	7	10
KMT2C	7	3
MAP3K1	6	10
GATA3	6	7
PTEN	5	5
SF3B1	4	2

**Table 2:** Most frequent clinically significant copy number variations (CNV) in DCIS and IBC

Top CNVs	DCIS Frequency (N)	IBC Frequency (N)
MCL1	15	15
CKS1B	12	13
ERBB2	9	5
JAK1	5	4
IKZF1	5	4
BCL11B	4	3
PAX5	4	4
HNF1B	4	4
KDM5C	4	1
APLNR	3	3
GATA1	3	4
WT1	3	2
ELF3	2	3
FAT1	2	2
FOXA1	2	0
PTPRT	1	2
AMER1	1	2

**Table 3:** Comparison of top clinically significant variants and copy number variations (CNVs) in IBC and DCIS. Each row represents a variant or copy number variation broken down into categories pertaining to which samples the alteration was present in. The “total” column represents the number of unique patients with that somatic mutation or copy number variation

Top Variants	Function	IBC only	DCIS only	In both	Total
PIK3CA	Gain	3	4	14	21
TP53	Loss	3	1	6	10
MAP3K1	Loss	4	1	4	9
GATA3	Gain	3	2	4	9
KMT2C	Loss	0	3	3	6
PTEN	Loss	1	2	2	5
SF3B1	Gain	0	2	2	4

Top CNVs	Function	IBC only	DCIS Only	In both	Total
MCL1	Gain	7	8	8	23
CKS1B	Gain	7	6	6	19
ERBB2	Gain	0	4	5	9
JAK1	Loss	3	4	1	8
IKZF1	Loss	2	3	2	7
BCL11B	Loss	2	3	1	6
PAX5	Loss	2	2	2	6
APLNR	Loss	3	3	0	6
KDM5C	Loss	1	4	0	5
GATA1	Loss	2	1	2	5
WT1	Loss	1	2	1	4
ELF3	Gain	2	1	1	4
FAT1	Loss	2	2	0	4
PTPRT	Loss	2	1	0	3
AMER1	Loss	2	1	0	3
HNF1B	Loss	1	1	1	3
HNF1B	Gain	0	0	2	2
FOXA1	Loss	0	1	0	1
FOXA1	Gain	0	1	0	1

### RNA-seq analysis

**Fig. 2:**
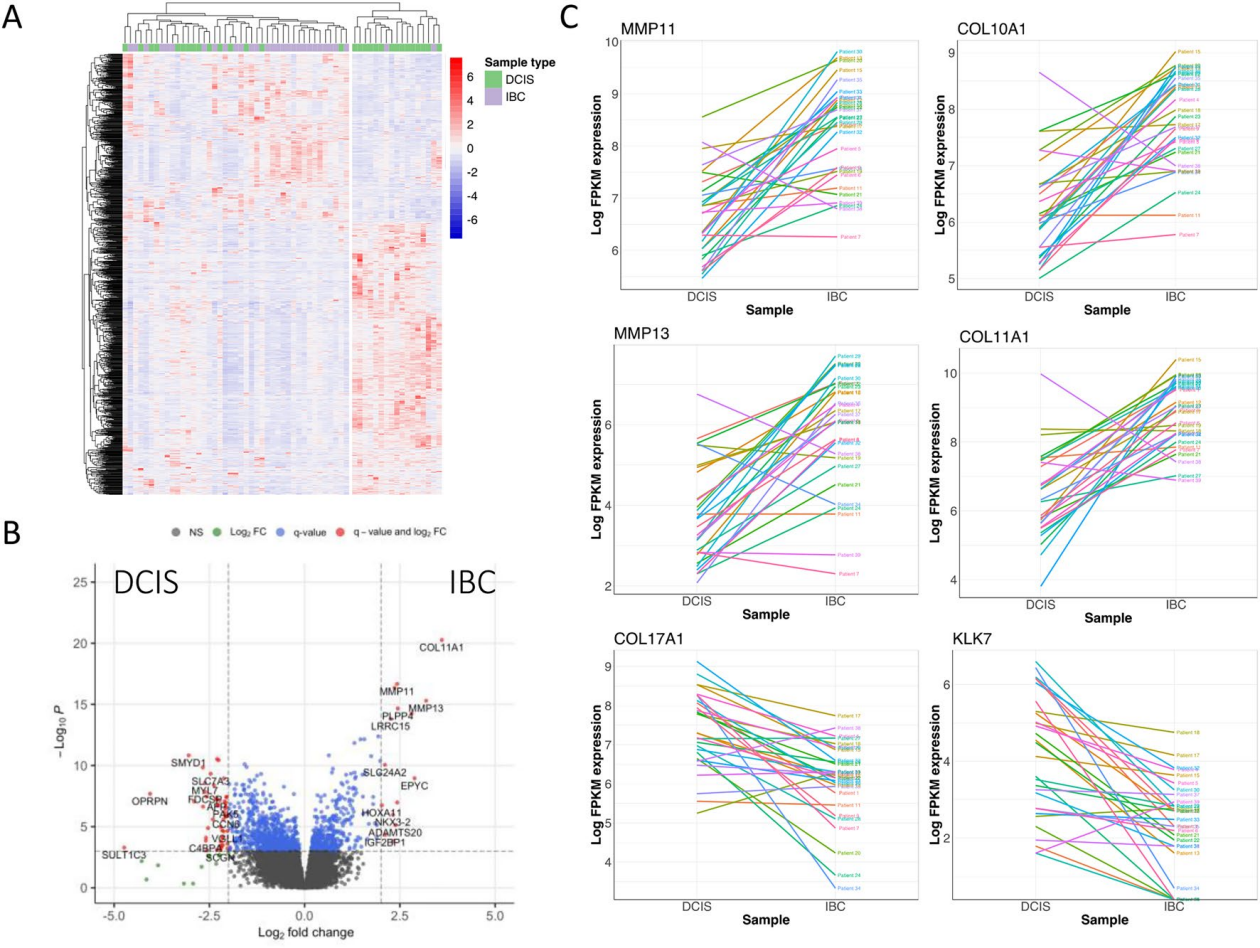
Differential expression of co-occurring DCIS versus IBC. **A** Heatmap of the 1209 genes that are significantly differentially expressed between DCIS and IBC (*q* < 0.001). **B** Volcano plot showing fold change versus -log_10_ p-value for the DCIS vs. IBC comparison. **C** Gene expression differences between DCIS vs IBC are shown for each patient for top DE genes plotted by log FPKM gene expression

We next asked whether there were significant gene expression differences between DCIS and IBC. Looking at the groupwise level, we found 1209 genes were significantly differentially expressed (DE) between DCIS and IBC (FDR-corrected *q*-value < 0.001) (Fig. 2 A&B and Table 4), with the top DE genes upregulated in IBC largely related to extracellular matrix formation and comprising collagen-related genes (*COL11A1, COL10A*), matrix metallopeptidase subunits (*MMP11, MMP13, MMP1,* etc.) as well as genes associated with microfibril assembly (*FN1, MFAP2, LRRC15,* etc.). Meanwhile, top genes specifically associated with DCIS were related to muscle cell regulation (*SMYD1, PDE1C, PAMR1*), olfactory receptors (*OR5P3, OR5P2*) and immune related functions (*IL33, LIFR,* etc.). We evaluated gene expression differences on a patient-by-patient basis for each of the top DE genes (Fig. 2C) and noted that the difference between expression for DCIS and IBC largely trended in the same direction for each patient (with a few exceptions). However the overall magnitude of the difference is variable across each patient.

**Table 4:** Top differentially expressed genes between DCIS and IBC (*q*-value < 0.001)

Gene name	Ensemble gene ID	Description	HGNC ID	Log2 fold change	Adjusted *p*-value	Directionality
COL11A1	ENSG00000060718.22	Collagen type XI alpha 1 chain	2186	3.530	4.37E-21	Enriched in IBC
MMP11	ENSG00000099953.10	Matrix metallopeptidase 11	7157	2.403	3.95E-18	Enriched in IBC
COL10A1	ENSG00000123500.10	Collagen type X alpha 1 chain	2185	2.296	1.36E-16	Enriched in IBC
MMP13	ENSG00000137745.13	Matrix metallopeptidase 13	7159	3.144	1.68E-16	Enriched in IBC
PLPP4	ENSG00000203805.11	Phospholipid phosphatase 4	23,531	2.384	5.76E-15	Enriched in IBC
LRRC15	ENSG00000172061.9	Leucine rich repeat containing 15	20,818	2.212	1.63E-14	Enriched in IBC
MMP1	ENSG00000196611.6	Matrix metallopeptidase 1	7155	2.704	6.05E-14	Enriched in IBC
FN1	ENSG00000115414.21	Fibronectin 1	3778	1.533	7.72E-13	Enriched in IBC
SYNDIG1	ENSG00000101463.6	Synapse differentiation inducing 1	15,885	1.883	8.43E-13	Enriched in IBC
INHBA	ENSG00000122641.11	Inhibin subunit beta A	6066	1.423	2.46E-12	Enriched in IBC
OVCH2	ENSG00000183378.13	Ovochymase 2	29,970	− 2.325	3.05E-12	Enriched in DCIS
SMYD1	ENSG00000115593.15	SET and MYND domain containing 1	20,986	− 3.008	4.93E-12	Enriched in DCIS
OR5P3	ENSG00000182334.3	olfactory receptor family 5 subfamily P member 3	14,784	− 2.257	6.54E-12	Enriched in DCIS
PDE1C	ENSG00000154678.18	Phosphodiesterase 1C	8776	− 1.577	1.42E-11	Enriched in DCIS
MFAP2	ENSG00000117122.14	Microfibril associated protein 2	7033	1.253	2.92E-11	Enriched in IBC
CD276	ENSG00000103855.18	CD276 molecule	19,137	0.618	3.03E-11	Enriched in IBC
KLK7	ENSG00000169035.12	Kallikrein related peptidase 7	6368	− 2.667	3.03E-11	Enriched in DCIS
IL33	ENSG00000137033.12	Interleukin 33	16,028	− 1.516	3.35E-11	Enriched in DCIS
ADAMTS7	ENSG00000136378.15	ADAM metallopeptidase w/thrombospondin type 1 motif 7	223	0.717	8.59E-11	Enriched in IBC
ST6GAL2	ENSG00000144057.16	ST6 beta-galactoside alpha-2,6-sialyltransferase 2	10,861	1.682	8.59E-11	Enriched in IBC
OLR1	ENSG00000173391.9	Oxidized low density lipoprotein receptor 1	8133	1.536	8.90E-11	Enriched in IBC
GJB2	ENSG00000165474.8	Gap junction protein beta 2	4284	1.915	1.14E-10	Enriched in IBC
SLC7A3	ENSG00000165349.12	Solute carrier family 7 member 3	11,061	− 2.469	1.16E-10	Enriched in DCIS
OR5P2	ENSG00000183303.3	Olfactory receptor family 5 subfamily P member 2	14,783	− 2.181	1.34E-10	Enriched in DCIS
SULF1	ENSG00000137573.14	Sulfatase 1	20,391	1.086	1.62E-10	Enriched in IBC
EPYC	ENSG00000083782.8	Epiphycan	3053	2.977	1.83E-10	Enriched in IBC
CEMIP	ENSG00000103888.17	Cell migration inducing hyaluronidase 1	29,213	1.962	1.83E-10	Enriched in IBC
UNC5B	ENSG00000107731.12	unc-5 netrin receptor B	12,568	1.201	1.83E-10	Enriched in IBC
ADAMTS14	ENSG00000138316.11	ADAM metallopeptidase w/thrombospondin type 1 motif 14	14,899	1.559	2.57E-10	Enriched in IBC
MYL7	ENSG00000106631.8	Myosin light chain 7	21,719	− 2.733	3.26E-10	Enriched in DCIS
PAMR1	ENSG00000149090.13	Peptidase domain containing associated w/muscle regeneration 1	24,554	− 1.228	4.15E-10	Enriched in DCIS
COL17A1	ENSG00000065618.21	Collagen type XVII alpha 1 chain	2194	− 1.837	4.15E-10	Enriched in DCIS
PLAU	ENSG00000122861.16	Plasminogen activator, urokinase	9052	1.313	4.36E-10	Enriched in IBC
LIFR	ENSG00000113594.10	LIF receptor subunit alpha	6597	− 1.115	4.89E-10	Enriched in DCIS
COL12A1	ENSG00000111799.22	Collagen type XII alpha 1 chain	2188	1.269	5.65E-10	Enriched in IBC
KRT17	ENSG00000128422.18	Keratin 17	6427	− 2.419	5.65E-10	Enriched in DCIS

**Fig. 3:**
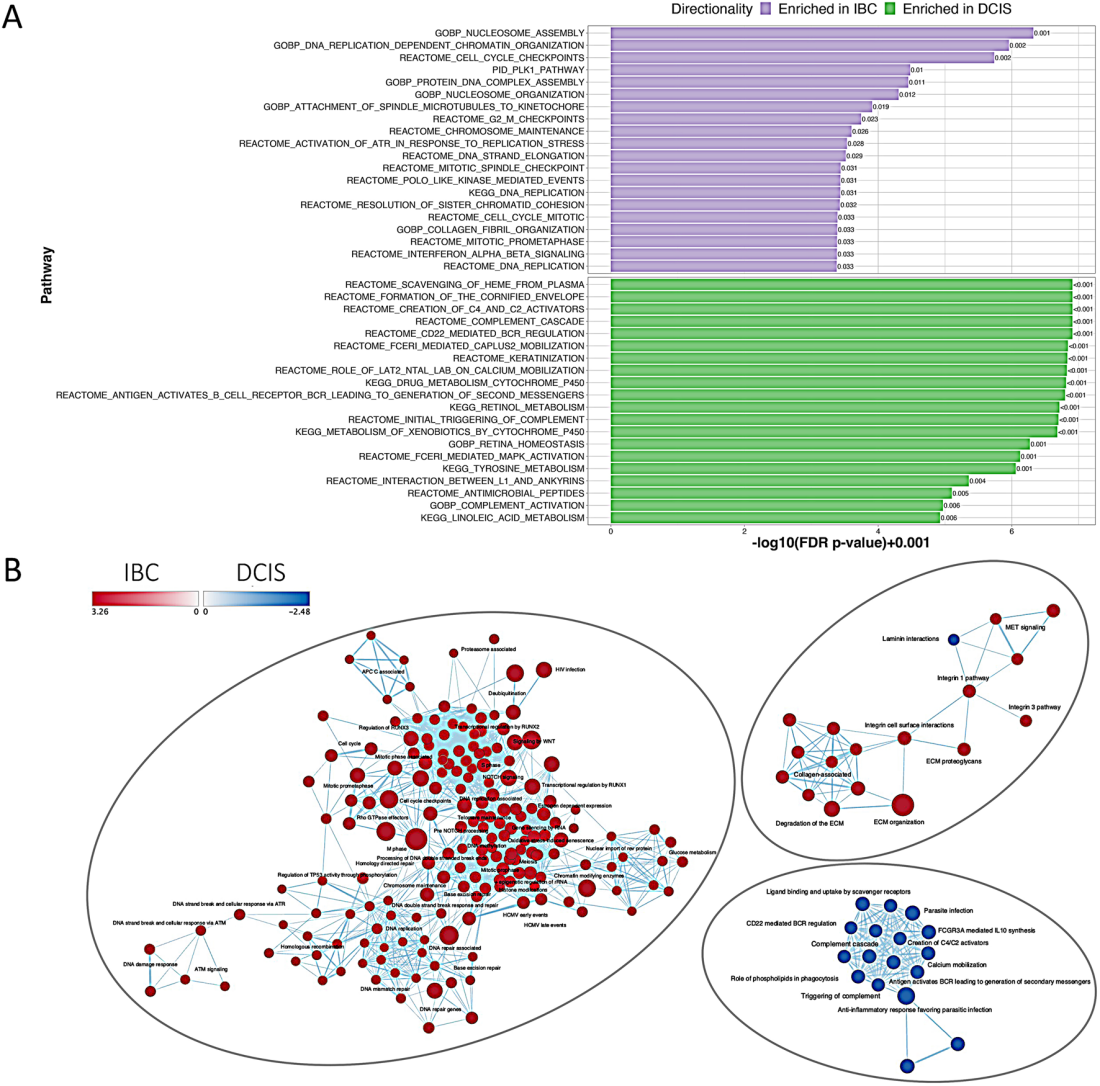
Ranked whole transcriptome level pathway enrichment for DCIS vs IBC. **A** Top 20 most significant pathways from the MSigDB C2 and C5 biological processes collections tested for pathway enrichment using DE genes from the DCIS vs IBC tumor contrast utilizing GSEA. **B** Cytoscape networks highlighting 3 major clusters from GSEA for DCIS vs IBC on MsigDB C2 all collection. The color of the node indicates the normalized enrichment score (NES) and directionality assigned by ranked GSEA

We then asked whether certain a priori cellular pathways were significantly enriched as DE between DCIS and IBC. To do this we performed pathway enrichment analysis using multiple approaches including Gene Set Enrichment Analysis (GSEA) across a variety of gene set collections as well as DAVID functional annotation for DE genes. The GSEA results weighing heavily on KEGG, Gene Ontology and Reactome pathways, yielded significant differences between DCIS and IBC (Fig. 3), with IBC samples significantly overexpressing pathways associated with cycling cells and proliferation (e.g. “Replication Dependent Chromatin Organization” (GO), “DNA Replication” (KEGG) etc.) but also pathways associated with replication stress and activation of cell cycle checkpoints (“Activation of ATR in Response to Replication Stress” (Reactome), “Cell Cycle Checkpoints” (Reactome), consistent with active proliferation and dysregulated DNA replication. For DCIS, many of the enriched pathways were related to cytochrome (CYP) enzymes and xenobiotic metabolism (“Metabolism of xenobiotics by cytochrome P450” (KEGG) and immune responses (“Complement Cascade” (Reactome), “B-Cell Receptor Signaling” (GO)). We also assessed enrichment of specific transcription factor binding sites (TFBS) with the associated DE genes and observed a variety of enriched TFBS (Figure S1). The top site enriched in the IBC was a promoter regulatory element of unknown function (M120 motif KRCTCNNNNMANAGC, *q* < 0.001) [37] as well as sites regulated by heat shock transcription factor 4 (HSF4) (*q* < 0.02). Top TFBS enriched in DCIS included MEF2 (*q* < 0.005), HMEF2 (*q* < 0.006), AR (*q* < 0.002) and CEBPE (*q* < 0.003).

**Fig. 4:**
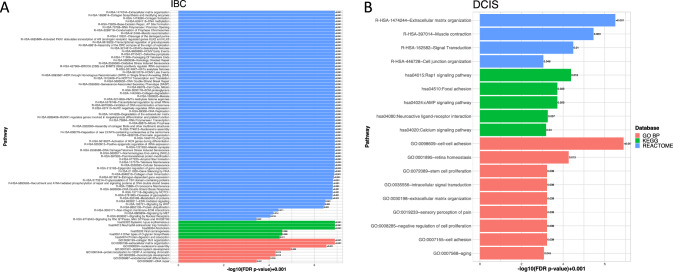
Pathway enrichment for DCIS vs IBC. **A** Top significant DAVID pathways from the Reactome, KEGG and Gene Ontology (Biological Processes only) collections based on differentially expressed genes enriched in IBC resulting from differential expression analysis between DCIS and IBC tumors. **B** Top significant pathways from DAVID involving DE genes enriched in DCIS tumors

**Fig. 5:**
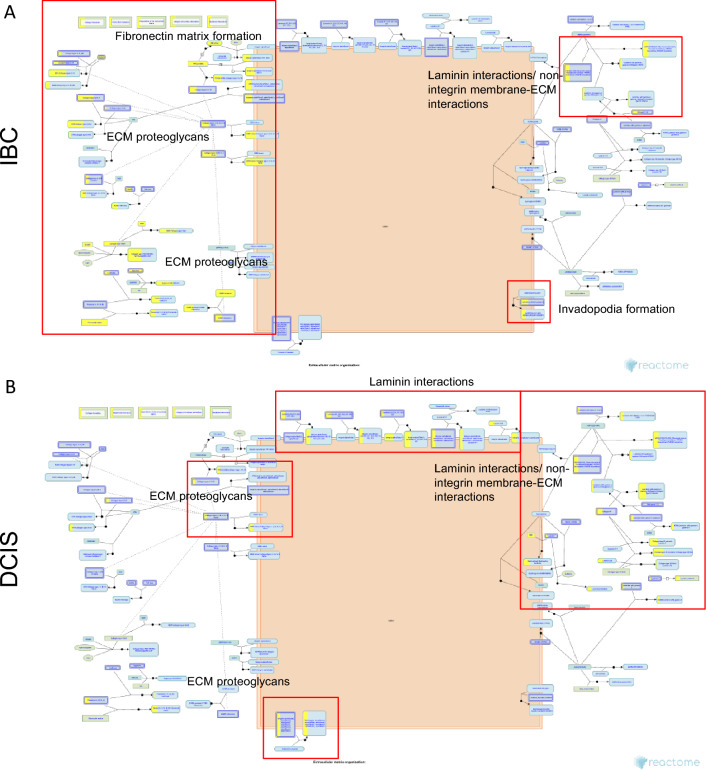
Differential expression of ECM regulatory networks by DCIS and IBC. **A** Genes overexpressed in IBC are highlighted in yellow overlayed on Reactome pathway R-HSA-1474244 ~ Extracellular matrix organization, with red boxes outlining key components of the ECM machinery enriched in IBC. **B** The same pathway diagram, with overexpressed genes in DCIS highlighted

We also tested for pathway enrichment using DAVID, which uses only the significant DE genes as opposed to GSEA (which uses a transcriptome-wide enrichment approach). While there was significant overlap between the two approaches, including significant enrichment for pathways associated with DNA replication and cell cycle progression in IBC, the top pathways associated with IBC using DAVID were related to ECM organization and regulation (Fig. 4A). Interestingly, the top pathways associated with DCIS also included several pathways associated with ECM organization (Fig. 4B), leading us to hypothesize that ECM organization is mechanistically different between DCIS and IBC. To further characterize these differences, we plotted genes differentially expressed in DCIS vs. IBC on Reactome pathway diagrams for ECM formation (Fig. 5A, B). There we observed distinct components of ECM formation and organization enriched in DCIS and IBC lesions, with invadopodia formation components and proteoglycans specifically enriched in IBC, while DCIS shows specific enrichment for integrin/laminin signaling (Fig. 5).

### Integration of DNA-seq and RNA-seq

**Fig. 6:**
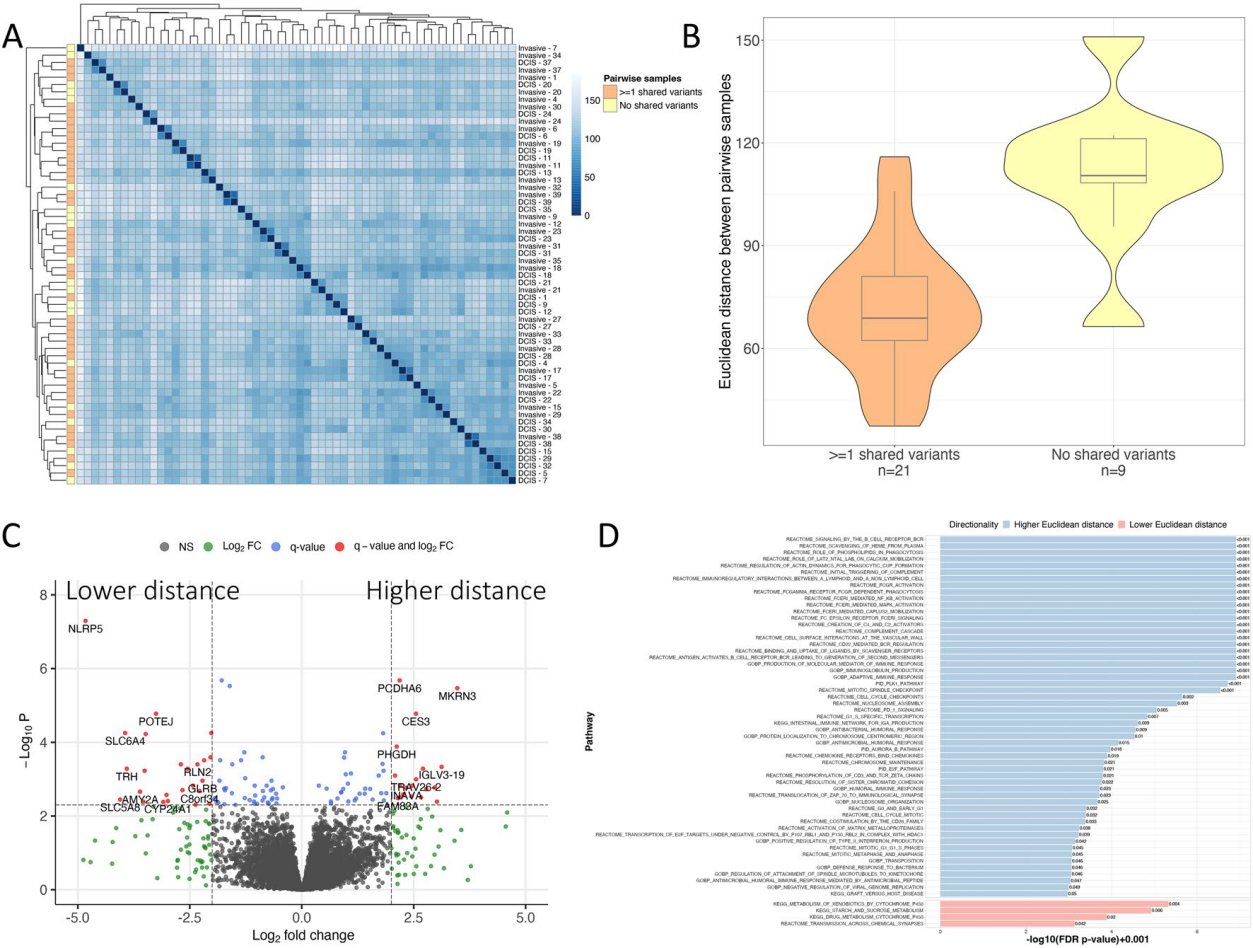
Transcriptome similarity and the association with shared genomic variants. **A** Sample-to-sample matrix of transcriptome similarity via Euclidean distance. **B** Violin plot and Student’s T-test between transcriptome-wide gene expression Euclidean distance of pairwise samples for patients whose paired DCIS/IBC shared genomic variants versus those that did not. **C** For tumors with shared variants, transcriptional differences between those with high and low gene expression similarity. **D** Pathway enrichment analysis of DE genes between patients who had high versus low gene expression similarity via GSEA

To characterize the concordance/discordance between DNA-seq and RNA-seq profiles observed across the DCIS and IBC lesions we constructed a similarity matrix using Euclidean distance for gene expression across DCIS and IBC profiles. We then asked whether DCIS/IBC pairs with shared mutations were more similar in gene expression patterns than DCIS/IBC pairs without shared mutations. We see that for the most part gene expression patterns are more similar between an individual’s paired DCIS/IBC lesions than with DCIS or IBC from another individual (Fig. 6A). Comparing patients with shared variants in their DCIS/IBC pairs versus those without shared variants, we observed that gene expression was significantly more diverse when DCIS and IBC had no shared genomic variants (Euclidean distance of overall gene expression between paired DCIS and IBC, *p* < 0.005) (Fig. 6B), likely more evidence that these lesions developed independently versus via a common progenitor. However, even within lesions that had highly similar mutational genomes, we observed a subset of DCIS/IBC pairs that had highly variable gene expression. To characterize factors driving this, we subdivided mutantnome-specific tumors into two groups based on higher or lower gene expression similarity. We detected significant expression differences between these groups (Fig. 6C), with divergent DCIS/IBC pairs showing upregulation of pathways associated with B-cell receptor signaling (*q* < 0.001), heme scavenging (*q* < 0.001) and phagocytosis (*q* < 0.001). Pairs with highly similar transcriptomes showed enrichment for a variety of metabolic pathways (Fig. 6D).

**Fig. 7:**
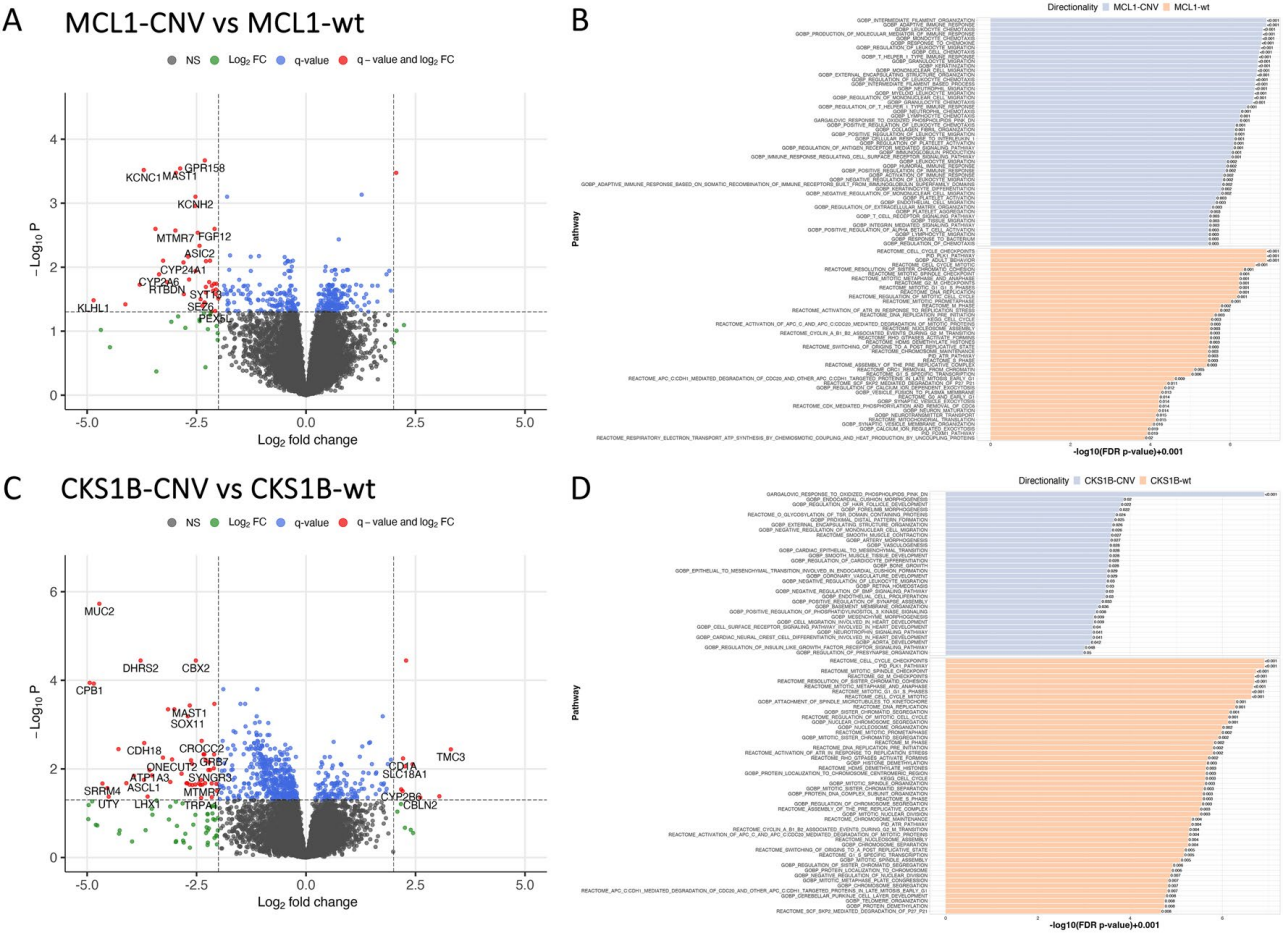
Correlation between copy number variations and transcriptional differences. **A** Volcano plot showing differential expression between *MCL1-CNV* versus *MCL1-wt* tumors. **B** Pathway enrichment analysis of gene expression differences between *MCL1-CNV* versus *MCL1-wt* tumors using GSEA and highlighting MSigDB C2 and C5 biological processes collections. **C** Volcano plot showing differential expression between *CKS1B-CNV* versus *CKS1B-wt* tumors. **D** Pathway enrichment analysis of gene expression differences between *CKS1B-CNV* versus *CKS1B-wt* tumors

We also asked whether significant mutational differences were associated with large-scale changes to the transcriptome. Given the frequency of *MCL1* copy number variations in breast cancer, we asked whether there were transcriptional differences between *MCL1-*amplified versus wildtype lesions (Fig. 7A). From this we observed 357 genes that were significantly different between *MCL1-CNV* and *MCL1-wt* lesions (*q* < 0.05) with key differences in extracellular matrix organization and adaptive immune response pathways (Fig. 7B). Similarly, we asked whether there were significant differences between tumors with *CKS1B* copy number variations versus *CKS1B* wildtype. From differential expression analysis, we observed 627 differentially expressed genes (*q* < 0.05) between *CKS1B-CNV* and *CKS1B-wt* lesions (Fig. 7C)*,* with the top targets related to endothelial function and angiogenesis (Fig. 7D). From these combined data, we conclude that co-occurring DCIS and IBC display a wide degree of heterogeneity across individuals, and within individuals there are varying degrees of heterogeneity that are correlated with specific somatic mutations and copy number variations.

Finally, we compared ER+ /PR+ to ER+ /PR-patients. Comparisons were made between these groups for IBC vs IBC, DCIS vs DCIS, and differences between paired IBC/DCIS patients for ER+ /PR+ vs ER+ /PR-cohorts. The only marked difference was the finding of overexpression of *ERBB2* in ER+ /PR-DCIS vs ER+ /PR+ DCIS (supplementary text). The number of ER+ /PR-patients is quite small in this series so the significance of these analyses requires confirmation in a larger cohort.

## Discussion

Co-occurring DCIS and IBC lesions continue to represent a compelling model for understanding the evolution of DCIS to IBC. There are many advantages versus other systems as it allows us to directly interrogate these lesions in humans as opposed to model organisms as well as look in a common genetic background (*i.e.* within an individual). Indeed, co-occurring DCIS/IBC lesions are typically more similar to each other based on gene expression than to other lesions with the same histological subtype such as DCIS versus DCIS, likely showing that the original genetic background is the key driver for the majority of basal transcription (Fig. 6), even showing greater similarity than for different cases with the same oncogenic driver alterations (Fig. 1). The most frequent genomic variants in both DCIS and IBC were noted to be *PIK3CA, TP53, KMT2C, MAP3K1, GATA3* and *SF3B1*, with *KMT2C* being more frequent in DCIS and *TP53* and *MAP3K1* more frequent in IBC, though the numbers are too small for definitive conclusions. The most frequent copy number variations were seen in *MCL1, CKSB1* and *ERBB2*. *ERBB2* changes were not seen in IBC unless present in the corresponding DCIS. As such, while there have been some advances in developing prognostic gene expression signatures for DCIS progression, differences in genetic background are likely a key factor currently limiting the power of such approaches [11, 38]. Studies involving significantly larger cohorts of DCIS patients with associated expression and outcome information will be key to fully account for these factors.

Overall, our data and that from others point to a model for co-occurring lesions where a subset of similar DCIS/IBC lesions seem to arise from a common somatic progenitor, while others with highly divergent mutational and expression profiles appear to arise mostly independently. Indeed, just under 20% of DCIS and IBC cases shared no variant or copy number changes. This finding is quite similar to that of Lips et al*.*, who examined such changes in a comparison of DCIS and later recurrence of invasive disease [22]. In that study approximately 25% of subsequent but temporally distinct invasive recurrences appeared to be clonally distinct from the original DCIS. These data are supportive of a more general “field effect” hypothesis throughout the breast rendering it susceptible to subsequent carcinogenesis. Our work extends this current literature in providing a combined portrait of gene expression and mutational changes across these co-occurring lesions. Common intuition would suggest that co-occurring DCIS/IBC with similar somatic genomes would appear more similar in their gene expression programs, and indeed they do in our dataset (Fig. 6). However, note that even in DCIS/IBC pairs with similar somatic genomes, there is a large spread in transcriptional similarity/dissimilarity. Looking more deeply at those with transcriptional dissimilarity (Fig. 6C), reveals that a large proportion of the detected variation comprises immune-related pathways (Fig. 6D). Thus, it is likely that these differences are not necessarily related to a more divergent tumor transcriptome but related to more immunogenicity in this subset of lesions driving the infiltration of diverse immune cell types. As such, this metric may be useful for predicting which subset of lesions may be responsive to immunotherapeutic treatment modalities.

While these expression data showed several key phenotypes distinct to IBC in relation to DCIS, one key mechanism that stood out was the significant difference in expression for key subsets of extracellular matrix formation and signaling. DCIS ECM expression was hallmarked by laminin/integrin signaling (laminin 322, etc.) as markers for early stage disease. In contrast, IBC ECM gene expression featured fibronectin matrix formation (Fig. 5), which is corroborated by recent mechanistic work by Hayward et al*.* who show that DCIS to IBC progression is associated with deposition of fibronectin in the duct and duct expansion potentially as a mechanism supporting invasion [39]. IBC also showed expression of invadopodia formation machinery, likely representing a key step in transitioning to a metastatic state. In addition, a large number of additional detected genes did not fit into known breast cancer progression mechanisms (*e.g. SYNDIG1, OVCH2, OR5P3,* etc.) and further studies of these targets may yield new insights into additional mechanisms that support breast cancer progression.

We also took an integrative look at somatic mutations and transcriptional activity across paired DCIS/IBC lesions (Fig. 7). Notably, we observed a transcriptional program that was distinct to *MCL1*-amplified tumors (Fig. 7A). We noted upregulation of pathways related to immune response, cell migration and activation, chemotaxis and apoptosis in DCIS and IBC samples with increased CNVs for the *MCL1* gene. Copy number variations for the *MCL1* gene, which has been implicated in apoptotic functions, have been reported in many hematologic as well as solid malignancies, including breast cancer [40–43]. *MCL1* copy number variations have been seen in triple negative, hormone receptor positive and *ERBB2*-positive breast cancer [40, 42–44] and have been correlated with both prognosis and resistance to various antitumor therapies [45, 46]. Campbell et al*.* examined *MCL1* in the MMTV-PyMT genetic mouse model of breast carcinogenesis, which recapitulates features of breast cancer progressing through hyperplasia to metastasis [47, 48]. This study revealed that deletion of *MCL1* in the mammary epithelium of the genetically engineered mice showed an “absolute requirement” for *MCL1* in breast tumorigenesis [47]. We are unaware of prior data on *MCL1* in preinvasive human breast cancer. While it is well established that *MCL1* is important in anti-apoptotic activities [49], the data presented here suggest the need for additional evaluation of its role in modulating the tumor immune microenvironment.

Unlike *MCL1*-amplified tumors, *CKS1B-*altered tumors showed differences related to endothelial function and angiogenesis (Fig. 7C, D). CKS1B protein is known to bind to the catalytic subunit of the cyclin-dependent kinases and has a major role in cell cycle regulation and has been implicated in apoptosis inhibition as well [50–53]. *CKS1B* amplification and overexpression have been reported in breast cancer and multiple myeloma and has been associated with poor prognosis in both [51, 54, 55]. Slotky et al*.* has reported *CKS1B* in breast cancer was associated with patient’s age, estrogen, and progesterone receptor levels and increased with malignant degree while Shi et al*.* has implicated it in the development of drug resistance [55, 56]. Jia et al*.* noted an association of *CKS1B* activity and infiltration of breast tumors with cancer-associated fibroblasts and noted and association with cell cycle and kinase regulation [51].

These data suggest additional areas of opportunity for studies to be performed. The changes in transcriptomics and pathway analysis can be confirmed at the proteomic level and/or using metabolomics to assess the metabolic output of these pathways. In particular, the differences in extracellular matrix activities noted in this study and others [38, 39] and immune-related phenomena between DCIS and IBC [17–19] may yield clues to the seminal events in the change from non-invasive to invasive activity. Spatial genomic studies of the tumor microenvironment may allow for the assessment of morphological changes in the ECM that result from these pathway alterations. It is clear that the genomic changes that are found in IBC are already often present in DCIS, leading to the question of when in the evolution from normal breast tissue to invasive cancer these events occur. Studies analogous to those presented here in patients with atypical ductal hyperplasia as well as histologically normal breast tissue in these patients would be helpful in this regard and would address the “field effect” hypothesis.

In addition, with larger patient cohorts, tumor-specific alterations in genomics, transcriptomics and proteomics can be correlated with clinical prognostic parameters, such as hormone receptor and *ERBB2* status, histologic and nuclear grade, Ki67 and clinically available genomic predictive assays such as OncotypeDx DCIS [57]. We hope this and other work may help usher in more clinically useful biomarkers and potential therapeutic targets to better predict and modify the evolution of these breast lesions.

## Conclusion

Overall, the genomic and transcriptomic characterization of co-occurring DCIS/IBC lesions shows a diverse spectrum of patterns spanning close co-evolution to clonally independent lesions. Moreover, the specific genetic backgrounds underlying these tumors drives complex transcriptomic programs that may have key relevance for progression and/or response to treatment. Even within these subgroups there is considerable diversity, suggesting epigenetic or other levels of regulation are additional key contributors to tumorigenesis that have gone unmapped. Adding additional levels of characterization such as whole genome bisulfite sequencing, ATAC-seq, proteomics, detailed immune profiling and more may shed new light on tumor progression and lead to superior prognostic and/or predictive biomarker signatures as well as lead to novel treatment paradigms.

### Supplementary Information

Below is the link to the electronic supplementary material.

Supplementary file1 (DOCX 2312 KB)

Supplementary file2 (DOCX 14 KB)

## Data Availability

The datasets generated during and /or analyzed during this study are available from the corresponding author on reasonable request.
