# Supplementary material for: Multi-omic profiling of simultaneous ductal carcinoma in situ and invasive breast cancer
Source: Breast Cancer Res Treat. 2024 Mar 24;205(3):451–64. doi: 10.1007/s10549-024-07270-5 (PMC11101558; doi:10.1007/s10549-024-07270-5)
Supplement: Supplementary file 1 — Supplementary file1 (DOCX 2312 KB) [file 10549_2024_7270_MOESM1_ESM.docx]

**Supplementary Text**

Multi-Omic Profiling of Simultaneous Ductal Carcinoma in situ and Invasive Breast Cancer

Henry G. Kaplan, MD^1,2,4^, Alexa K. Dowdell, MS^3,4^, Anna B. Berry, MD^1^, Racheli Ben Shimol, BSc^3^, Fred L. Robinson, PhD^3^, Christopher A. Carney, BS^1^ and Brian D. Piening, PhD^3^

Corresponding Author: Henry G. Kaplan – [hank.kaplan@swedish.org](mailto:hank.kaplan@swedish.org)

Breast Cancer Research and Treatment

**Figure S1.** **Pathway enrichment involving transcription factor targets for DCIS vs IBC.** A. Pathway analysis of gene expression differences between DCIS and IBC against MSigDB C3 transcription factor targets collection for GSEA. B. Top significant pathways with directionality indicating enrichment in either DCIS or IBC (q<0.05).

**ER+PR+ IBC vs. ER+PR- IBC**

The underpowered test resulted in 87 differentially expressed (DE) genes at q<0.05. 45 DE genes were enriched in ER+PR+ IBC and 42 DE genes were enriched in ER+PR- IBC samples (Figure S2A&B). Pathway enrichment analysis via ranked GSEA on a curated subset of C2 all containing a majority of KEGG, Reactome, and Gene Ontology pathways as well as C5 biological processes (bp) revealed ER+PR+ IBC tumors enriched in SRP dependent cotranslational protein targeting to membrane (q<0.008), eukaryotic translation initiation (q<0.01) and selenoamino acid metabolism (q<0.026). ER+PR- IBC tumors were only enriched in one pathway being evaluated at an FDR-corrected p-value of 0.1, which was homophilic cell adhesion via plasma membrane adhesion molecules (q<0.001) (Figure S2C).


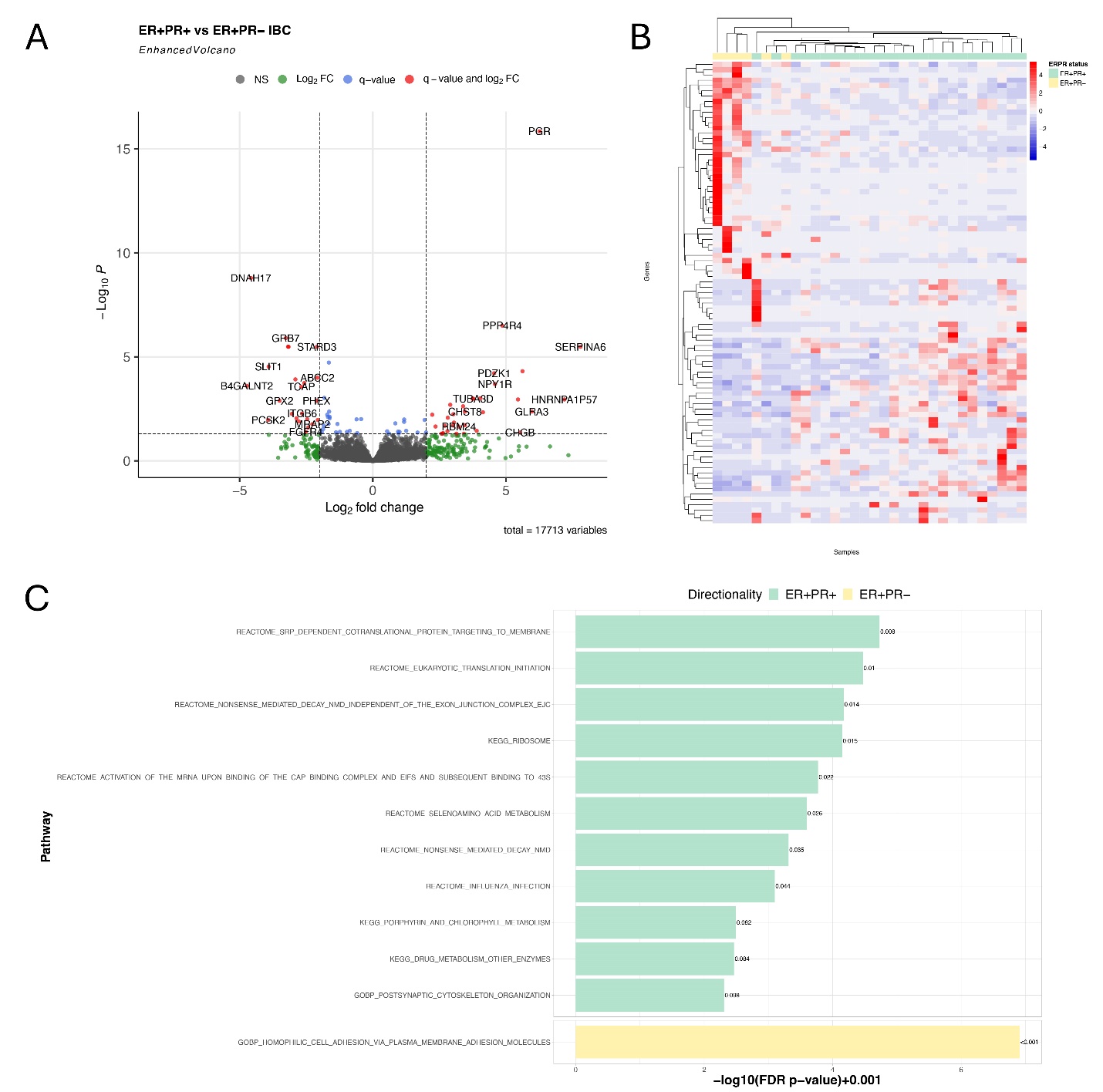


**Figure S2.** **ER+PR+ vs ER+PR- differentially expressed genes and pathway enrichment in IBC tumors.** A. Volcano plot of differentially expressed genes between ER+PR+ IBC and ER+PR- IBC (q<0.05). B. Heatmap showing patterns of differentially expressed genes between ER+PR+ IBC and ER+PR- IBC (q<0.05). C. Pathway analysis of gene expression differences between ER+PR+ IBC and ER+PR- IBC against MSigDB C2 subset, and C5 biological processes collections for ranked GSEA. Top significant pathways with directionality indicating enrichment in either ER+PR+ or ER+PR- IBC (q<0.1).

**ER+PR+ DCIS vs. ER+PR- DCIS**

Differential expression analysis resulted in 665 DE genes at q<0.05 (Figure S3A&B). 394 genes were differentially expressed in ER+PR+ DCIS samples, while 271 DE genes were overexpressed in ER+PR- DCIS including ERBB2 at q<0.0000000169.

Pathway enrichment analysis via ranked GSEA on a curated C2 all subset enriched for GO, KEGG, and Reactome and C5 bp revealed obvious trends of chemotaxis (neutrophil, lymphocyte, leukocyte, granulocyte, monocyte chemotaxis q<0.001), cell activation and migration (lymphocyte migration (q<0.001), neutrophil migration (q<0.001), b cell activation (q<0.001), t cell migration (q<0.001), regulation of t cell activation (q=0.004), regulation of leukocyte migration (q=0.002)) in ER+PR- DCIS samples (Figure S3C). We also observed trends of cell proliferation (leukocyte proliferation (q=0.001), b cell proliferation (q=0.002) and regulation of t cell proliferation (q=0.002), cell signaling (antigen receptor mediated signaling pathway (q<0.001) and immune response regulating cell surface receptor signaling pathway (q<0.001)), and immunity (leukocyte mediated immunity (q<0.001), lymphocyte mediated immunity (q<0.001), b cell mediated immunity (q<0.001),) in ER+PR- DCIS samples.


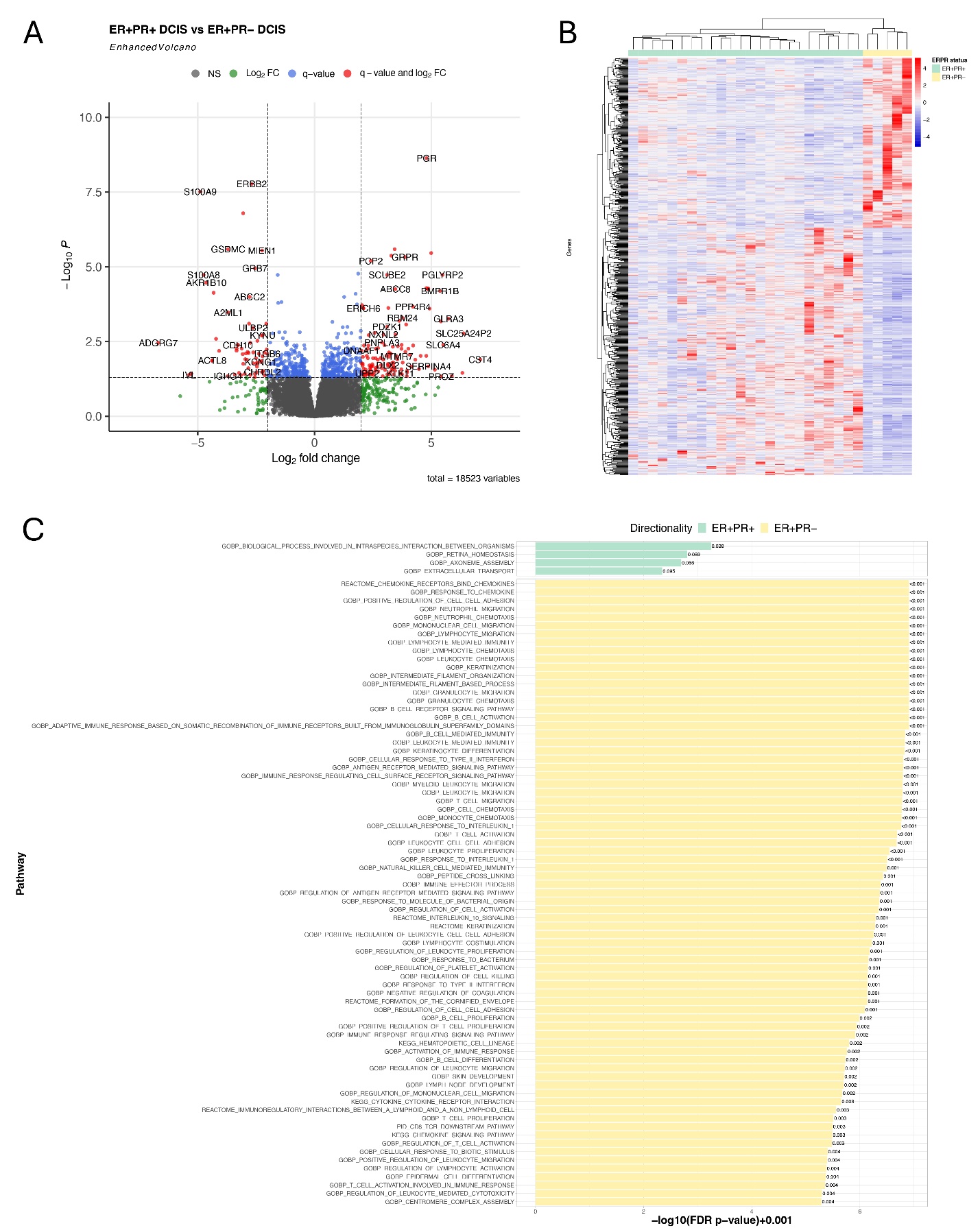


**Figure S3.** **ER+PR+ vs ER+PR- differentially expressed genes and pathway enrichment in DCIS samples.** A. Volcano plot of differentially expressed genes between ER+PR+ DCIS and ER+PR- DCIS (q<0.05). B. Heatmap showing patterns of differentially expressed genes between ER+PR+ DCIS and ER+PR- DCIS (q<0.05). C. Pathway analysis of gene expression differences between ER+PR+ DCIS and ER+PR- DCIS against MSigDB C2 subset, and C5 biological processes collections for ranked GSEA. Top significant pathways with directionality indicating enrichment in either ER+PR+ or ER+PR- DCIS (q<0.1).

**ER+PR+ paired IBC vs. DCIS**

Differential expression analysis on the paired ER+PR+ IBC tumors versus ER+PR+ DCIS samples resulted in 472 DE genes at q<0.001 while controlling for individual patient level differences in the comparison (Figure S4A). An FDR-corrected p-values of 0.001 was chosen to maintain consistency with the original pairwise IBC versus DCIS contrast. We observed 216 DE genes upregulated in ER+PR+ IBC samples and 256 DE genes in ER+PR+ DCIS samples. Top differentially expressed genes in ER+PR+ IBC tumors included MMP11, COL11A1, MMP1, MMP13 and COL10A1. ER+PR+ DCIS samples were overexpressed for OPRPN, CCN6, SMYD1, and RFX6. Pathway enrichment analysis via ranked GSEA resulted in numerous pathways associated with cell cycling (cell cycle mitotic (q<0.001) and cell cycle checkpoints (q<0.001)), DNA replication (q<0.001), chromosome separation (q<0.001) and collagen fibril organization (q<0.001) in ER+PR+ IBC (Figure S4B). ER+PR+ DCIS samples were associated with pathways involved in keratinization (q=0.015), the complement pathway (complement cascade (q<0.001) and creation of C4 and C2 activators (q<0.001)) and interestingly muscle contraction (q=0.02). Plotted are the top 30 significant pathways in each direction evaluated at q<0.05 (Figure S4B).


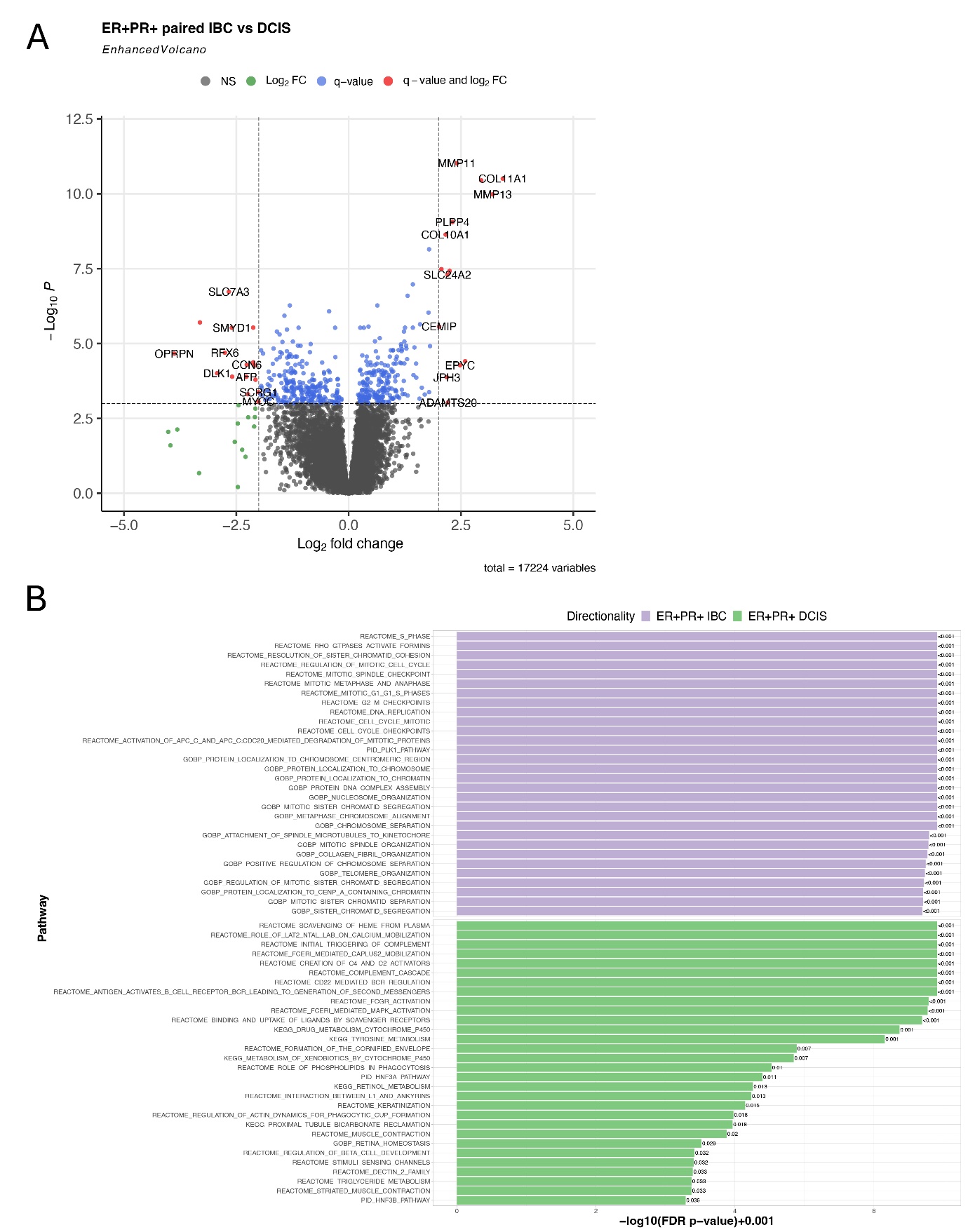


**Figure S4. Differentially expressed genes and pathway enrichment in ER+PR+ paired IBC versus DCIS samples.** A. Differentially expressed genes between paired ER+PR+ IBC and DCIS samples (q<0.001). B. Pathway analysis of gene expression differences between paired ER+PR+ IBC and DCIS against MSigDB C2 subset, and C5 biological processes collections for ranked GSEA. Top significant pathways with directionality indicating enrichment in either ER+PR+ IBC vs DCIS (q<0.05).

**ER+PR- paired IBC vs. DCIS**

Similarly, differential expression analysis was conducted pairwise on ER+PR- IBC tumors versus ER+PR- DCIS samples while controlling for patient level differences in the contrast. At an FDR-corrected p-value of less than 0.001, there were 195 DE genes between ER+PR- IBC vs DCIS with 57 genes and 138 genes overexpressed in ER+PR- IBC and ER+PR- DCIS, respectively (Figure S5A). Top differentially expressed genes in ER+PR- IBC are consistent with those up in ER+PR+ IBC including MMP11, COL11A1, MMP13, COL10A1 as well as COL12A1. Top differentially expressed genes in ER+PR- DCIS samples include PAX5, CCL19, MYL7. Pathway enrichment analysis via ranked GSEA was perhaps limited by the number of differentially expressed genes regardless of the ranked transcriptome approach (Figure S5B). ER+PR- IBC samples only had one significantly enriched pathway at q<0.05, collagen fibril organization (q=0.004) which overlaps with ER+PR+ IBC enriched pathway. Enriched pathways associated with ER+PR- DCIS include keratinization (q<0.001), the complement pathway (complement cascade (q<0.001) and creation of C4 and C2 activators (q<0.001)), similarly to pathways enriched in ER+PR+ DCIS samples**.** ER+PR- samples were also enriched in pathways consistent in ER+PR+ DCIS vs ER+PR- DCIS contrast including cell migration (t cell migration (q<0.001), neutrophil migration (q<0.001), and mononuclear cell migration (q<0.001)), chemotaxis (response to chemokine (q<0.001), neutrophil, lymphocyte and leukocyte chemotaxis (q<0.001)). Also of interest was ER+PR- DCIS pathway enrichment related to immune response (humoral immune response (q<0.001) and adaptive immune response (q<0.001)).


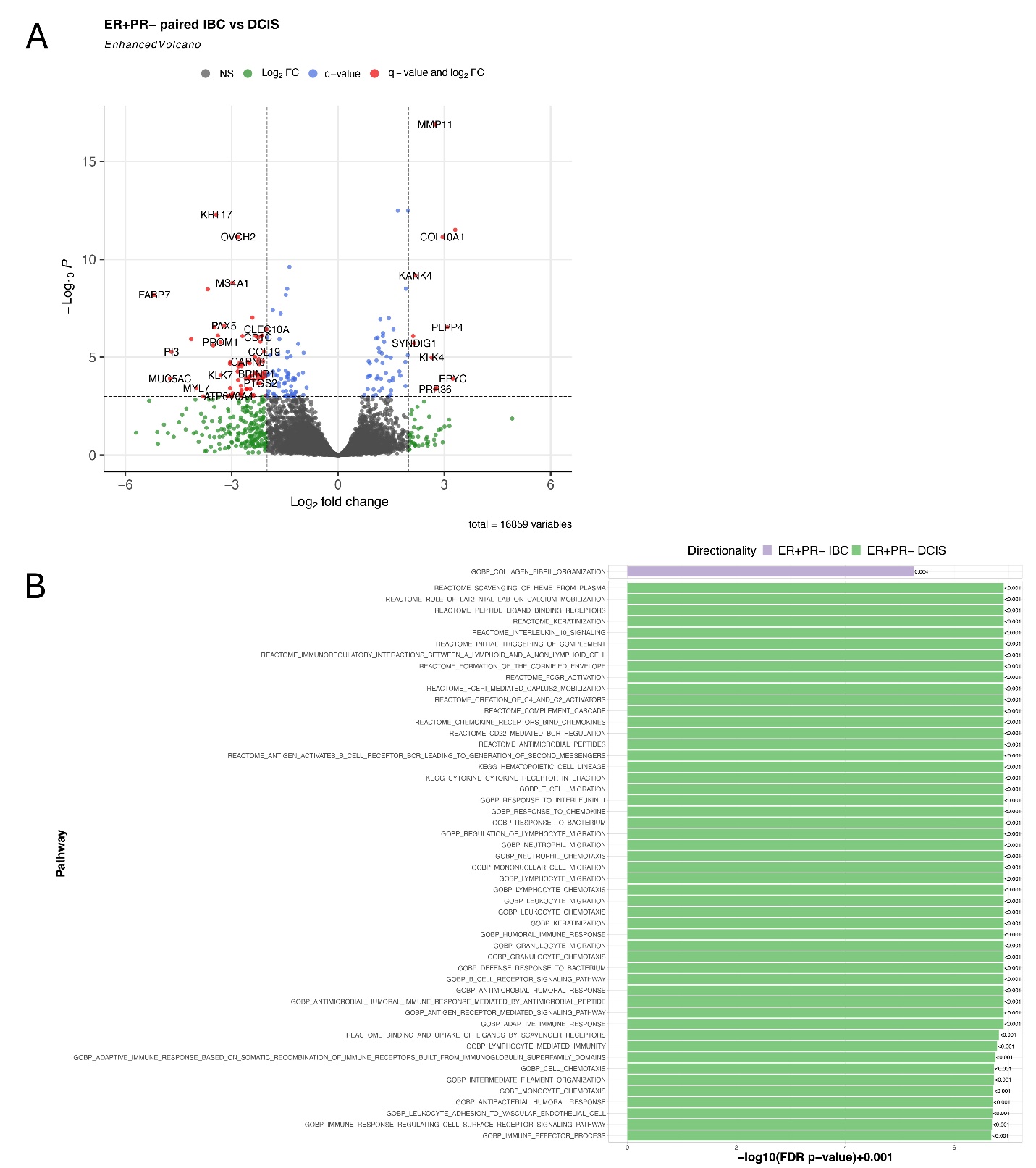


**Figure S5. Differentially expressed genes and pathway enrichment in ER+PR- paired IBC versus DCIS samples.** A. Differentially expressed genes between paired ER+PR- IBC and DCIS samples (q<0.001). B. Pathway analysis of gene expression differences between paired ER+PR- IBC and DCIS against MSigDB C2 subset, and C5 biological processes collections for ranked GSEA. Top significant pathways with directionality indicating enrichment in either ER+PR- IBC vs DCIS (q<0.05).

**Overlapping DE genes between ER+PR+ and ER+PR- pairwise (IBC vs DCIS) comparisons**

There were 39 differentially expressed genes upregulated in IBC that were overlapping between ER+PR+ and ER+PR- after comparing the resulting genes from the pairwise IBC vs DCIS contrasts (Figure S6A).

The 39 genes include: MMP11, COL11A1, MMP1, MMP13, PLPP4, COL10A1, LRRC15, INHBA, MFAP2, SYNDIG1, ST6GAL2, CEMIP, UNC5B, OLR1, CSMD2, SALL4, MMP14, NREP, KIF26B, COL1A2, ADAMTS16, SULF1, EPYC, COL5A2, COL12A1, KLK4, NOX4, FNDC1, FN1, BGN, SGIP1, COL8A1, AEBP1, SUGCT, ITGA11, ENSG00000130635.16, ENSG00000249406.3, ENSG00000168542.16, ENSG00000183098.11.

We observed 28 differentially expressed genes upregulated in DCIS that were overlapping between the ER+PR+ and ER+PR- pairwise contrast for IBC vs DCIS samples (Figure S6B).

The 28 overlapping genes include: IL33, LIFR, OR5P3, PDE1C, OVCH2, SYNM, KLK7, CAPN6, ALPL, APOD, OR5P2, TRPM6, HAS3, ACTG2, SELP, MYL7, STAC2, ABCB1, FDCSP, KRT5, OSR1, KRT14, KRT17, SFRP1, ENSG00000145934.16, ENSG00000112936.19, ENSG00000003096.14, ENSG00000105894.12.

**Figure S6. Overlapping differentially expressed (DE) genes between paired ER+PR+ IBC vs DCIS and ER+PR- IBC vs DCIS samples.** A. Overlap and set difference between pairwise ER+PR+ IBC DE genes and ER+PR- IBC DE genes (q<0.001). B. Overlap and set difference between pairwise ER+PR+ DCIS DE genes and ER+PR- DCIS DE genes (q<0.001).
