# Supplementary material for: Multi-omic profiling of simultaneous ductal carcinoma in situ and invasive breast cancer
Source: Breast Cancer Res Treat. 2024 Mar 24;205(3):451–64. doi: 10.1007/s10549-024-07270-5 (PMC11101558; doi:10.1007/s10549-024-07270-5)
Supplement: Supplementary file 2 — Supplementary file2 (DOCX 14 KB) [file 10549_2024_7270_MOESM2_ESM.docx]

| **Pathway** | **Size** | **NES** | **p value** | **FDR q value** | **Directionality** |
| --- | --- | --- | --- | --- | --- |
| **KRCTCNNNNMANAGC_UNKNOWN** | 58 | 2.727 | 0.000 | 0.000 | Enriched in IBC |
| **MEF2_01** | 117 | -1.844 | 0.000 | 0.005 | Enriched in DCIS |
| **HMEF2_Q6** | 108 | -1.827 | 0.000 | 0.050 | Enriched in DCIS |
| **HSF4_TARGET_GENES** | 15 | 2.228 | 0.000 | 0.019 | Enriched in IBC |
| **AR_03** | 53 | -1.694 | 0.001 | 0.043 | Enriched in DCIS |
